# Anesthesia-induced developmental neurotoxicity in the setting of systemic inflammation: the role of microglia

**DOI:** 10.3389/ebm.2025.10549

**Published:** 2025-05-16

**Authors:** Nemanja Useinovic, Adre Newson, Michelle Near, Stefan Maksimovic, Benjamin Volvovitz, Nidia Quillinan, Vesna Jevtovic-Todorovic

**Affiliations:** ^1^ Department of Anesthesiology, University of Colorado Anschutz Medical Campus, Aurora, CO, United States; ^2^ Neuronal Injury and Plasticity Program, University of Colorado Anschutz Medical Campus, Aurora, CO, United States

**Keywords:** tibial fracture, neonatal rats, sevoflurane, lipopolysaccharide, microglia, neuroinflammation, developmental neurotoxicity

## Abstract

Although it is well documented in animal research that an early exposure to general anesthetics during critical stages of synaptogenesis disturbs normal brain development ultimately leading to cognitive and affective impairments, it is less clear whether and how surgical interventions and/or underlying systemic inflammation impact the detrimental effects of general anesthetics. Some emerging evidence suggests that aseptic systemic inflammation preceding exposure to the commonly used general anesthetics worsens anesthesia-induced neuroapoptosis and activates inflammasome pathways while resulting in impaired cognitive-affective behaviors. To improve our understanding of the underlying mechanisms, here we focused on multicellular interactions between damaged neurons and microglia since microglia is the resident macrophages within the brain that respond to stress. Using infant rats (post-natal day 7) and most commonly used inhaled anesthetic, sevoflurane, we examine microglia role in sevoflurane-induced inflammation-propagated developmental neurotoxicity. We show that sevoflurane exposure leads to a significant neuroapoptosis in young rat pup hippocampal subiculum, a neuroapoptosis that is worsened in the setting of systemic inflammation caused by either lipopolysaccharide (LPS) injection or trauma (tibial fracture). The worsening is not only shown in terms of the intensity of neuroapoptosis but in its duration and onset. We further report that sevoflurane-induced neuroapoptosis triggers activation of microglia, which in turn releases proinflammatory cytokine MCP-1 and upregulates endothelial cell adhesion molecule, ICAM-1. This leads to T-lymphocyte infiltration in the hippocampal subiculum, an event that further perpetuates microglia activation in an attempt to control neuroapoptosis which is suggested by the fact that microglia depletion leads to a significant worsening of sevoflurane-induced developmental neuroapoptosis. Our work gets us a step closer to making our animal work more relevant to the clinical setting and hence more translational. This is vitally important considering that exposure to anesthesia is exceedingly rare in the absence of any kind of a pathological process.

## Impact statement

Over four million children are exposed to general anesthesia annually in the US alone. The work over the past couple of decades suggests that anesthesia exposure of a developing young brain may result in neuroapoptosis and long-lasting impairments of socio-cognitive development. Since exposure seldom occurs in the absence of an underlying disease and/or inflammation, the question remains whether and how this may affect anesthesia-induced developmental neurotoxicity. Our work and the work of others would suggest that disease-associated inflammation worsens neurotoxicity. Understanding the underlying mechanisms is crucially important in the field of clinical anesthesiology and fundamental science alike. We believe that our investigation of the role of microglia and their interplay with T-lymphocyte in the setting of sevoflurane-induced inflammation-propagated neuronal damage gets us a step closer to better understanding this phenomenon, hence allowing us to develop promising strategies for the use of general anesthesia in a true disease setting.

## Introduction

Based on numerous preclinical and emerging clinical findings over the past couple of decades, it is becoming apparent that an early exposure to general anesthetics (GAs) during critical stages of synaptogenesis disturbs normal brain development ultimately leading to cognitive and affective impairments [1–9]. Preclinical studies thus far have aimed to understand this worrisome phenomenon by performing studies with GAs largely in isolation from underlying disease processes that necessitate GA exposure in the first place. While this strategy had been extremely valuable in mechanistic analyses of GA-induced neurotoxicity, it has been repeatedly criticized. This is understandable since there is a significant gap in our understanding as to whether and how surgical interventions and/or underlying systemic inflammation impact the detrimental effects of GAs, especially considering that general anesthesia without an underlying illness or trauma is rare. In fact, systemic inflammation is prevalent in many disease processes and, importantly, surgical interventions themselves can initiate aseptic inflammatory responses [10, 11].

The work presented herein builds on our recently published observations that aseptic systemic inflammation preceding exposure to the commonly used inhaled GA sevoflurane, worsens sevoflurane-induced neuroapoptosis and activates inflammasome pathways while resulting in impaired cognitive-affective behaviors [12]. Here we focus on multicellular interactions between damaged neurons and microglia since microglia is the resident macrophages within the brain that respond to stress, typically triggering a pro-inflammatory state [13–16]. Hence, we set out to examine their role in several aspects of sevoflurane-induced inflammation-propagated developmental neurotoxicity. Our interest in the interactions between neurons and microglia is based on the fact that brain is considered an immune privileged organ lacking circulating immune cells [17, 18].

In the context of neuronal injury, damage associated molecular patterns (DAMPs) released by dying neurons trigger pro-inflammatory microglial activation and release of cytokines, in particular monocyte chemoattractant protein-1 (MCP-1) among others [19–22]. Since we have previously reported an increase in sevoflurane-induced neuroapoptosis in the setting of systemic inflammation [12], the question we asked here was whether infiltrating immune cells contribute to ongoing inflammatory processes and hence, whether they play a detrimental role in herein presented ongoing and enhanced neuronal destruction long after the offending agents (sevoflurane and biochemical products of inflammation) are no longer present. Furthermore, we also examine the expression of intercellular adhesion molecule-1 (ICAM-1) that enables peripheral immune cells to enter the brain [23] and assess the role of immune cell brain infiltration.

We introduce two disease-type systemic inflammatory states in neonatal rodents at peak of their brain development (post-natal day 7): lipopolysaccharide (LPS) injection and a novel neonatal tibial fracture model of trauma, each initiated prior to sevoflurane exposure thus enabling us to focus on the role of preemptive systemic inflammation.

In this study we first confirm that systemic inflammation enhances sevoflurane-induced neuroapoptosis and report that this is accompanied by heightened activation of microglia, upregulation of cytokine, MCP-1which is known to upregulate cell adhesion molecules that allow peripheral immune cells to enter the brain [21, 24] and increased expression of ICAM-1, a cell adhesion molecule and transmembrane glycoprotein that could be induced by upregulated MCP-1. We also report a role of microglia in subsequent T-lymphocyte infiltration in subiculum of sevoflurane-exposed animals in the setting of systemic inflammation.

## Materials and methods

### Ethical statement

All experimental procedures were approved by the Institutional Animal Care and Use Committee of the University of Colorado Anschutz Medical Campus and adhered to the NIH Guide for the Care and Use of Laboratory animals. All efforts were made to minimize the number of animals used and procedures were performed in full compliance with Public Health Service’s Policy on Humane Care and Use of Laboratory Animals.

### Animals

Sprague-Dawley dams were purchased with PND 4-5 pups of equal gender (Envigo, Indianapolis, IN, United States), and housed under a 14/10 light-dark cycle with *ad libitum* access to food and water until pups were appropriate age for experiments. Upon arrival, animals were allowed a minimum of 36 h acclimation period prior to use.

### Tibial fracture

PND6 rat pups were anesthetized with 3% sevoflurane. Animals were transferred to surgical station pre-heated to 37°C, and anesthesia was maintained with 2-3% sevoflurane administered via a nose cone. Animals were positioned supine, and the entire body was covered with sterile drapes except the right lower extremity. Skin was disinfected with iodine and alcohol swabs hip to ankle.

Under sterile conditions, the knee of the lower right extremity was flexed to expose the tibial plateau and patellar ligament. Stainless steel 0.38 mm inner rod of 22-gauge Quincke style spinal needle (McKesson, Irving, TX, United States) was advanced through the tibial medullary canal from the superomedial aspect of the tibial plateau to 1 cm above the ankle joint. The trajectory of the needle was parallel to the tibial shaft, 1-2 mm medial to the patellar ligament projection.

Upon secure placement, the proximal end of the needle was clipped off at the entry point using wire cutters. A small skin incision was made on the medial surface of the tibia midway along the shaft to expose the underlying bone. Transverse fracture was produced using sterilized straight Bonn scissors (Fine Science Tools, Foster City, CA, United States). Fracture point was confirmed, and stability verified with gentle manual manipulation of proximal and distal fragments. External pressure was applied to achieve hemostasis, and skin was reapproximated and sealed with veterinarian glue. Neosporin ointment was applied to spinal needle entry point as well as the skin incision site. Animal was disconnected from the anesthesia and placed on a heated blanket until they regained consciousness and were able to remain sternal. Animal was subsequently returned to the home cage and reunion with the dam closely observed over 30 min for signs of distress such as hypomobility, nursing difficulties, symptoms of agitation and unusual vocalization.

Sham surgery counterparts, randomly selected within the same litters, were similarly induced with 3% sevoflurane and transferred to the surgical station. Animals were kept under anesthesia for a duration of time equivalent to the average procedure time of the surgery animals within the litter. During this time, small incision was made mid-tibia medially, and soft tissues were gently manipulated with sterile tools to emulate injury of surrounding soft tissue. Neither intramedullary fixation nor sharp tibial fracture were performed. Skin incision was closed with veterinary glue, and animals were returned to the home cage and observed as described for fracture procedure.

### LPS treatment

Lipopolysaccharide (LPS) model of systemic inflammation was induced as previously described [12]. Briefly, LPS (*E. coli*, O55:B5) was purchased from Sigma Aldrich (St. Louis, MO, United States) and dissolved in 1x phosphate buffered saline (PBS). On PND6, animals were randomly assigned to receive 1 mg/kg LPS intraperitoneally (i.p.) or PBS vehicle.

### Microglial manipulations

Minocycline hydrochloride was purchased from Sigma Aldrich (St. Louis, MO, United States). PLX-5622 was purchased from Cayman Chemical (Ann Arbor, MI, United States). Immediately prior to injection, minocycline was dissolved in warm PBS, whereas PLX-5622 was dissolved in 10% dimethyl sulfoxide (DMSO) and 25% β-cyclodextrin (Santa Cruz Biotechnology, Santa Cruz, CA, United States).

To inhibit microglial activation, animals were randomly administered two i.p. doses of minocycline 40 mg/kg, based on the published literature [25, 26], or PBS vehicle. The first loading dose was administered 1 h prior to LPS, and second given 12 h later, 1 h before anesthesia exposure. To deplete microglia from the developing brain, 65 mg/kg PLX-5622 or DMSO-cyclodextrin vehicle were administered i.p. in 3 doses 24 h apart on PND4, 5 and 6, as determined by our pilot study.

### Anesthesia exposure

Twelve hours after the tibial fracture or LPS injection, animals now at PND7 were exposed to 3% sevoflurane administered in 30% oxygen carrier gas or 30% oxygen carrier gas only for 3 hours, as described in our previous study [12]. In addition to being relevant in dose and duration [27, 28], this anesthesia regimen proved to be reliable and reproducible method in our previous study of inducing neuroapoptosis in PND7 rat pups [12]. Ambient temperature of 35.5°C and gas concentrations (oxygen, CO_2_ and sevoflurane) were continuously monitored in real time for the duration of the exposure (Datex Ohmeda Capnomac Ultima, Helsinki, Finland). Heart rate, blood glucose and oxygen measurements were obtained at the end of the 3 h exposure period in a subset of animals. Left ventricular blood sample was transcardially obtained for arterial blood gas analyses (i-STAT 1 Analyzer, Abbott Laboratories, Abbott Park, IL, United States) in a dedicated cohort, and given the terminal nature of the procedure, these animals were not used for further data analysis.

### Tissue collection

Euthanasia and tissue collection were performed at predetermined timepoints (1 h, 2 h, and 8 h) following sevoflurane exposure. For histological evaluation, animals were deeply anesthetized with isoflurane and transcardially perfused with ice-cold PBS followed by 4% paraformaldehyde (PFA). Brains were extracted and placed in 4% PFA overnight for post-fixation, then transferred to PBS + 0.025% sodium-azide and stored at 4°C.

For gene expression assays, animals were deeply anesthetized with isoflurane and brains rapidly extracted on ice. Left and right hippocampi were combined and flash-frozen in liquid nitrogen, then stored at −80°C prior to further processing.

### Neuroapoptosis quantification

Activated caspase-3 staining was performed as previously described [12]. Briefly, vibratome-cut sections were mounted on a slide, washed, quenched by submersion in Bloxall (Vector Labs, Newark, CA, United States) for 10 min and then blocked with 5% normal goat serum for 1 h at room temperature prior to staining. Stained serial 50 µm thick coronal brain slices (n = 4 subicula per animal) corresponding to −5.80 and −6.04 mm from bregma [29] were examined under Nikon Eclipse E800 microscope (Nikon Instruments, Melville, NY, United States). Images were obtained using a Nikon DS-Fi3 camera under a ×10 objective. Stained profiles were manually counted using a 500 µm^2^ grid placed over the regions of interest by an investigator blinded to treatment condition and multiplied by 2 to obtain the number of positive immunoprofiles per mm^2^, using NIS-Elements BR-5.11.02 software (Tokyo, Japan).

### Immunofluorescence staining and analysis

For microglial visualization, free floating sections (n = 4 subicula per animal) were washed three times in PBS+0.1% Triton X-100. Slices were blocked in 5% normal donkey serum (NDS) (Sigma Aldrich, St. Louis, MO, United States) for 1 h at room temperature, then incubated overnight at 4°C with goat anti-rat Iba1 primary antibody (1:500; Abcam, Cambridge, UK, ab5076). The following day, slices were washed three times with PBS+0.1% Triton X-100, then incubated with Alexa Fluor 488 donkey anti-goat secondary antibody (1:500, Jackson ImmunoResearch, West Grove, PA, United States, 705-545-003) for 2 h at room temperature protected from light. Slices were then washed three times, mounted, coverslipped and kept at 4°C until imaging.

High resolution confocal images were obtained under ×10 objective on Olympus FV-1200 confocal laser scanning microscope (Olympus Corporation, Tokyo, Japan). An investigator blinded to treatment conditions performed qualitative analysis of microglial morphology. Microglia were deemed inactive if they exhibited small somas and long branching processes, whereas activated state was identified by substantial soma enlargement and retraction of processes. Percent activation was calculated as a proportion of activated to the total number of microglia in the rat subicula per visual field, multiplied by 100 and averaged across all slices per individual animal.

### RNA extraction and quantitative polymerase chain reaction

Whole left and right hippocampus of each animal were combined, and total RNA isolated using RNeasy mini kit (Qiagen, Germany) according to the manufacturer’s instructions. 1,000 ng of RNA was used for cDNA synthesis (iScript Advance cDNA kit, Bio-rad, CA, United States). Quantitative real-time PCR was performed on a CFX Connect Real time system (Bio-rad, CA, United States) using SsoAdvanced Universal Probe Supermix (Bio-rad, CA, United States) and TaqMan gene expression assay probes for MCP-1 and ICAM-1 (Thermo Fisher Scientific, Waltham, MA, United States). Each sample was done in triplicate and normalized to the levels of GAPDH as a housekeeping gene. Results were expressed as fold changes compared to controls, which were assigned value of 1.

### Flow cytometry

Two hours after exposure, freshly harvested hippocampi were finely minced and incubated in calcium- and magnesium-free Hanks Buffered Salt Solution (HBSS) with addition of collagenase/dispase digestive enzymes (Sigma Aldrich, St. Louis, MO, United States) for 30 min at 37°C. Digested sample was diluted with excess HBSS, passed through 100 μm cell strainer, and pelleted in refrigerated centrifuge at 500 × g for 7 min. Pellet was resuspended in 70/30 Cytiva Percoll gradient centrifugation media (Thermo Fischer Scientific, Waltham, MA, United States) and centrifuged at 800 × g for 30 min with deceleration brakes disengaged. Interphase layer was collected, resuspended in HBSS and centrifuged at 500 × g for 7 min to wash away the Percoll. Single cell suspension was obtained by resuspending the pellet in 250 µL flow cytometry buffer.

All antibodies and dyes were purchased from BioLegend (San Diego, CA, United States). Staining steps were performed at 4°C. Cells and appropriate single-stain and fluorescence-minus-one (FMO) controls were blocked with anti-CD16/32 antibody (1:100) for 5 min, then incubated for 30 min with mastermix staining solution containing following antibody-fluorochrome products: ZombieYellow viability dye (1:1800), CD45-Pacific Blue (1:400), CD3-APC (1:100), B220-PE (1:200). Cells were then washed, resuspended in flow cytometry buffer and analyzed immediately on Gallios 561 flow cytometer (Beckmann Coulter, Brea, CA, United States). Briefly, single cells were identified using scatter features, live cells identified with viability dye, and CD45^+^ cells were gated then further separated into T-lymphocyte and B-lymphocyte populations.

### Statistical analysis

Statistical analysis was performed in GraphPad Prism 9.3. Differences between groups were assessed by the ANOVA followed by Tukey’s or Sidak’s *post hoc* test for one-way or two-way analyses, respectively. *α* was set at 0.05, thus *p*-values < 0.05 were considered statistically significant. Data were graphed as mean ± SEM, and level of significance indicated by elbow connectors with asterisks. For each dataset, males and females were separated and compared within each treatment group to establish the effect of sex differences. Analysis was performed using Two-way ANOVA and Sidak’s *post hoc*. Since we found no sex differences, the final set of data combined both sexes in each treatment group.

## Results

Based on our previous published work, it has been suggested that systemic inflammation results in worsening of sevoflurane-induced neuroapoptosis and upregulation of cytokines responsible for inflammasome activation [12]. Here we set out to further explore the severity of anesthesia-induced developmental neurotoxicity in the disease setting where we focus on a couple of timepoints of neuroapoptosis post-sevoflurane exposure as well as the role of microglia and cell adhesion molecules which allow for infiltration of circulating immune cells into the brain [21, 23, 24].

To continue to examine GA-induced inflammation-propagated developmental neurotoxicity we used two disease models: 1) systemic LPS injection and 2) trauma (tibia fracture as described in the Methods). The neurotoxicity was examined using activated caspase-3 (AC3+) staining as a reliable marker of neuroapoptosis. Our focus was on hippocampal subiculum for two main reasons: hippocampal subiculum is exquisitely sensitive to anesthesia-induced neurotoxicity [6, 30] and, as an extension of CA1 hippocampus, it plays an important role in socio-emotional development found to be impaired after an early exposure to anesthesia [2, 3, 31–34].

To assess the stability of relevant biological variables we carefully monitored and continuously recorded sevoflurane concentration, ambient temperature, oxygen delivery and CO_2_ concentration in anesthesia chamber to confirm the setting as stipulated in the Methods section. In addition, vital signs such as heart rate and oxygen saturation (using non-invasive pulse oximetry - SpO_2_) and blood glucose level were assessed in each animal group (control, LPS alone, sevoflurane alone and LPS + sevo). As shown in Table 1 we found no major changes in blood glucose concentration and SpO_2_ between groups when recorded at the end of sevoflurane exposure with an expected decrease in heart rate [35] in sevo and LPS + sevo groups. In addition, we studied arterial blood chemistry in a subset of animals within each cohort (sample sizes are included in Table 2). When collected and analyzed at the end of a 3-h sevoflurane exposure we confirmed that arterial blood chemistry, which reliably captures disturbances in tissue perfusion and oxygenation, was comparable to controls in each of the three experimental groups (Table 2).

**TABLE 1:** Blood glucose concentration, hemoglobin saturation and heart rate in each animal group.

	Control (n = 6) PBS injection 30% oxygen for 3 h	LPS (n = 6) 1 mg/kg LPS 30% oxygen for 3 h	Sevo (n = 6) PBS injection 3% sevo for 3 h	LPS + Sevo (n = 5) 1 mg/kg LPS 3% sevo for 3 h
Glucose (mg/dL)	98.17 ± 32.88	64.83 ± 14.22	115.3 ± 7.257	77.00 ± 36.38
SpO_2_ (%)	98.83 ± 0.4082	97.50 ± 1.975	95.00 ± 2.828	93.60 ± 2.608
Heart rate (per min)	343.5 ± 44.93	312.2 ± 15.35	217.5 ± 57.36 (***)	221.0 ± 34.27 (***)

Statistically significant differences in means are indicated in bold letters. PBS, phosphate buffered saline; LPS, lipopolysaccharide; SpO_2_, saturation of peripheral blood oxygen. ***p < 0.001.

**TABLE 2:** Arterial blood chemistry in each animal group.

	Control (n = 8) PBS injection 30% oxygen for 3 h	LPS (n = 9) 1 mg/kg LPS 30% oxygen for 3 h	Sevo (n = 8) PBS injection 3% sevo for 3 h	LPS + Sevo (n = 10) 1 mg/kg LPS 3% sevo for 3 h
pH	7.33 ± 0.05	7.32 ± 0.05	7.30 ± 0.06	7.30 ± 0.11
SpO_2_ (%)	95.50 ± 2.13	97.00 ± 0.76	93.22 ± 2.33	93.90 ± 3.57
paO_2_ (mmHg)	88.13 ± 9.77	98.50 ± 10.23	77.89 ± 5.64	81.90 ± 8.99
pCO_2_ (mmHg)	57.56 ± 6.72	47.68 ± 6.82	64.50 ± 8.88	49.50 ± 12.96

PBS, phosphate buffered saline; pH, potential of hydrogen; paO_2_, partial pressure of arterial oxygen; paCO_2_, partial pressure of arterial carbon dioxide; SpO_2_, saturation of peripheral oxygen.

**FIGURE 1:**
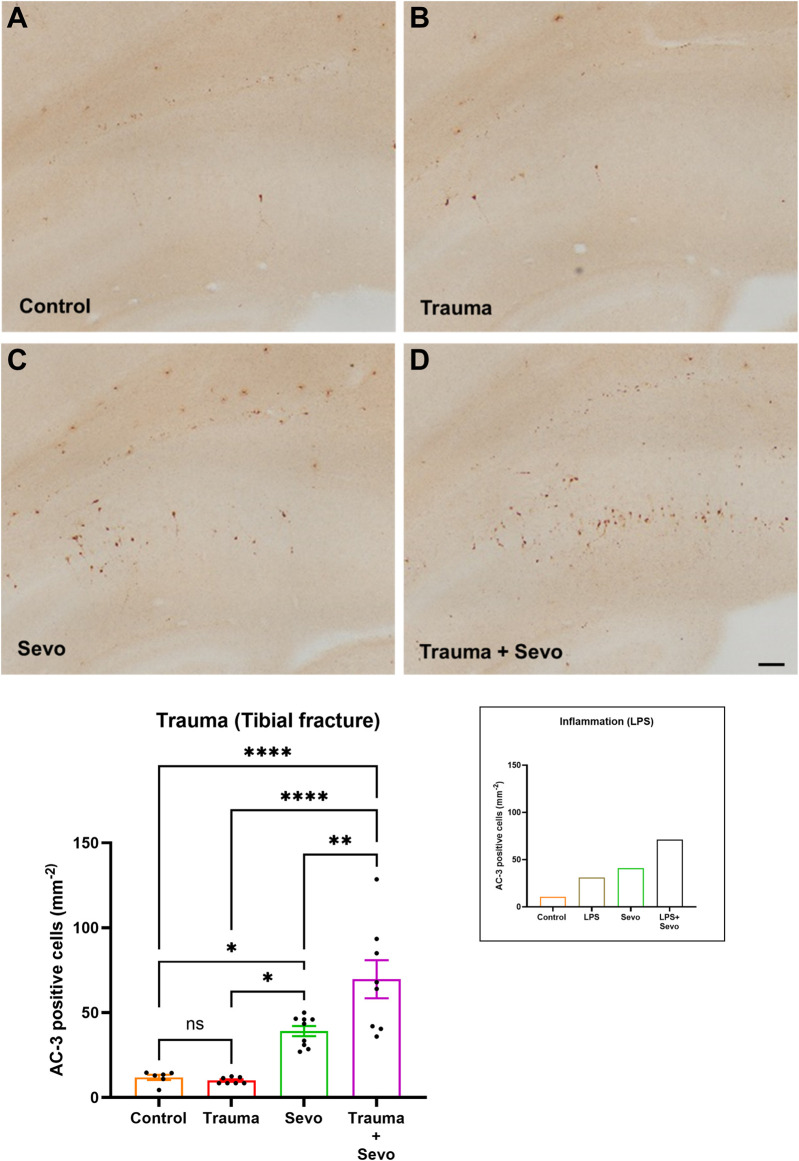
Preceding tibial fracture worsens sevoflurane-induced neuroapoptosis in the hippocampal subiculum of PND7 rat pups. Histomorphological analyses of neuronal apoptosis in the hippocampal subiculum are shown in the representative images of activated caspase-3 (AC-3^+^) staining in the hippocampal subiculum in control **(A)**, trauma **(B)**, sevoflurane **(C)** and trauma + sevoflurane **(D)** groups. The number of AC-3^+^ cells was increased in trauma + sevoflurane group compared with controls. ×10 magnification, scale bar is 100 μm. Bar graphs show the quantification analysis of AC-3^+^ positive cells per square millimeter in the hippocampal subiculum. Although trauma alone was indistinguishable from the control group, 3 hours of sevoflurane exposure significantly increased AC-3+cells compared with the control or trauma group. However, sevoflurane exposure in the setting of trauma (trauma + sevo group) the number of AC-3^+^ cells were greater compared with either treatment alone. Because there were no gender differences in AC-3^+^ cells densities, the results from both sexes were combined. For the ease of side-by-side comparisons, we include shadow bar graph in the upper right corner that shows previously published effects of lipopolysaccharide (LPS) on sevo-(sevoflurane) induced developmental neuroapoptosis. [12]. One-way ANOVA with Tukey’s *post hoc*. ns - non-significant, *p < 0.05, **p < 0.01, ****p < 0.0001.

We then proceeded to perform histomorphological analysis of developmental neuroapoptosis in each animal group (Figure 1A–D). As shown in Figure 1 we found that both LPS-induced systemic inflammation (previously reported [12] and shown here as a shadow graph in right upper corner) and trauma cause significant increase in activated caspase-3 positive cells (AC3^+^) in the developing subiculum of the animals exposed to sevoflurane for 3 hours compared to either sevoflurane alone or LPS/trauma alone. Interestingly, unlike LPS, trauma alone did not result in upregulated AC3^+^ cells when compared to sham controls. However, preemptive systemic inflammation caused by either LPS or trauma (p < 0.01) worsens developmental neuroapoptosis in young hippocampal subiculum of the animals exposed to sevoflurane at the peak of their synaptogenesis (PND7) compared to sevoflurane alone group.

**FIGURE 2:**
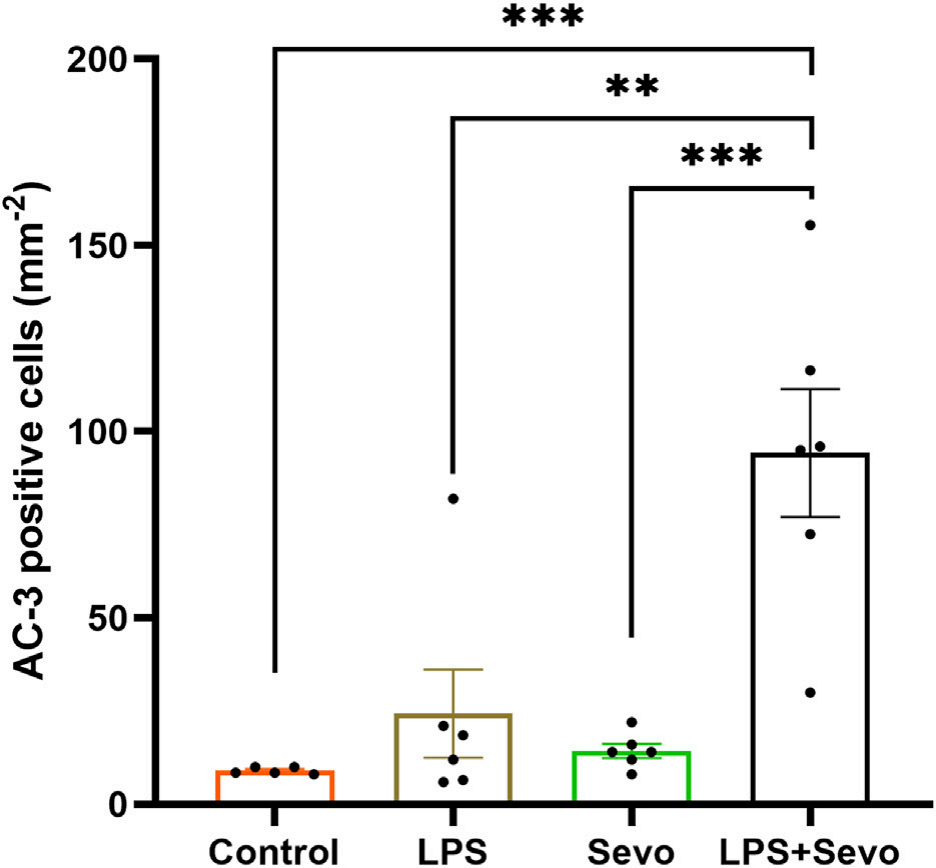
Short (1 hour) sevoflurane exposure in the setting of systemic inflammation induces significant neuroapoptosis in the hippocampal subiculum of PND7 rat pups. Quantitative analyses of AC-3^+^ positive cells per square millimeter in the hippocampal subiculum showed that a short exposure to sevoflurane alone is similar to controls, whereas the AC3^+^ level in sevoflurane group in the setting of LPS-induced systemic inflammation (LPS + sevo group) is significantly upregulated when compared to sham controls or the other two experimental groups. Because there were no gender difference in AC-3^+^ cells densities, the results from both sexes are combined. One-way ANOVA with Tukey’s *post hoc*. **p < 0.01, ***p < 0.001.

To assess the severity of GA-induced inflammation-propagated developmental neurotoxicity we set out to further examine the importance of inflammatory priming. Hence, we shortened the exposure to sevoflurane from 3 h (as shown in Figure 1) to only 1 h (Figure 2). We noted that although such short exposure has no bearing on AC3^+^ levels in sevoflurane alone groups when compared to controls, the AC3^+^ levels in sevoflurane group in the setting of LPS-induced systemic inflammation are significantly upregulated when compared to sham controls or the other two experimental groups (sevoflurane, p < 0.001 or LPS alone, p < 0.01) (Figure 2). Importantly, a short, 1-h sevoflurane exposure in the setting of inflammation is found to be as harmful as a 3-h exposure in the setting of systemic inflammation (Figure 2).

**FIGURE 3:**
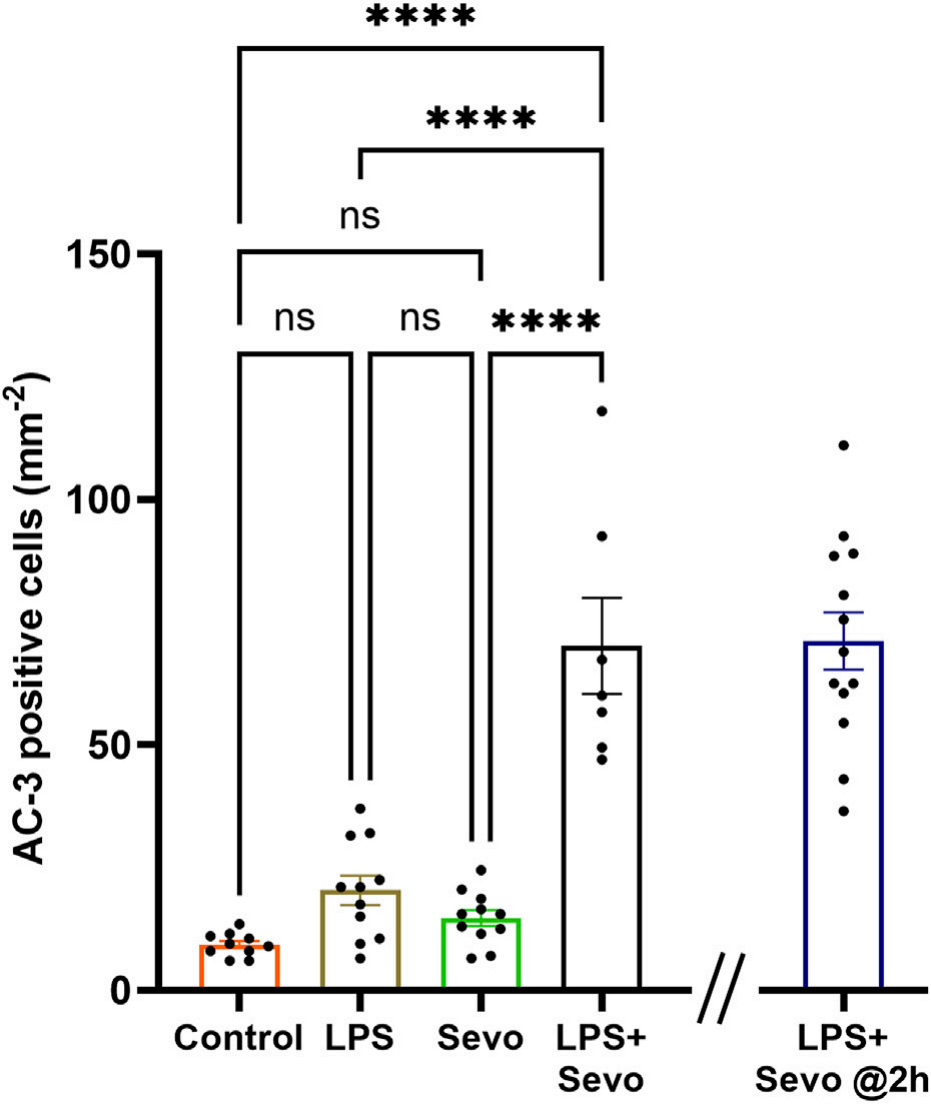
Sevoflurane exposure in the setting of LPS-induced systemic inflammation causes prolonged neuroapoptosis in the hippocampal subiculum of PND7 rat pups. Quantitative analyses of AC-3^+^ positive cells per square millimeter in hippocampal subiculum showed that the AC3^+^ levels in the LPS and sevoflurane alone groups at 8 h post-exposure are back to the control levels whereas the AC3^+^ levels in sevo-treated groups in the setting of systemic inflammation (LPS + sevo group) remain significantly upregulated when compared to either control, LPS or sevoflurane alone groups. The bar to the far right shows that the AC3^+^ levels in LPS + sevo group are of the similar magnitude at 8 h post-sevoflurane exposure as the one detected at 2 h post-sevoflurane exposure. Because there were no gender difference in AC-3^+^ cells densities, the results from both sexes are combined. One-way ANOVA with Tukey’s *post hoc*. ns - non-significant, ****p < 0.0001.

Furthermore, when we examined the delayed signs of neuroapoptosis, we found that the AC3^+^ levels in the LPS and sevoflurane alone groups at 8 h post-exposure are back to the control levels whereas the AC3^+^ levels in sevo-treated groups in the setting of systemic inflammation (LPS + sevo group) remain significantly upregulated when compared to either control, LPS or sevoflurane alone groups (p < 0.0001) (Figure 3). Interestingly, an ongoing neuroapoptosis in LPS + sevo group appears to be of the same intensity at 8 h timepoint post-sevoflurane exposure for 3 hours, as the one detected at the earlier timepoint, 2 h post-sevoflurane exposure (also shown in Figure 1). In aggregate, these findings suggest that the interactions between the inflammatory processes and general anesthesia result in amplified and prolonged neuronal demise hours after the offending agent (sevoflurane) is discontinued.

**FIGURE 4:**
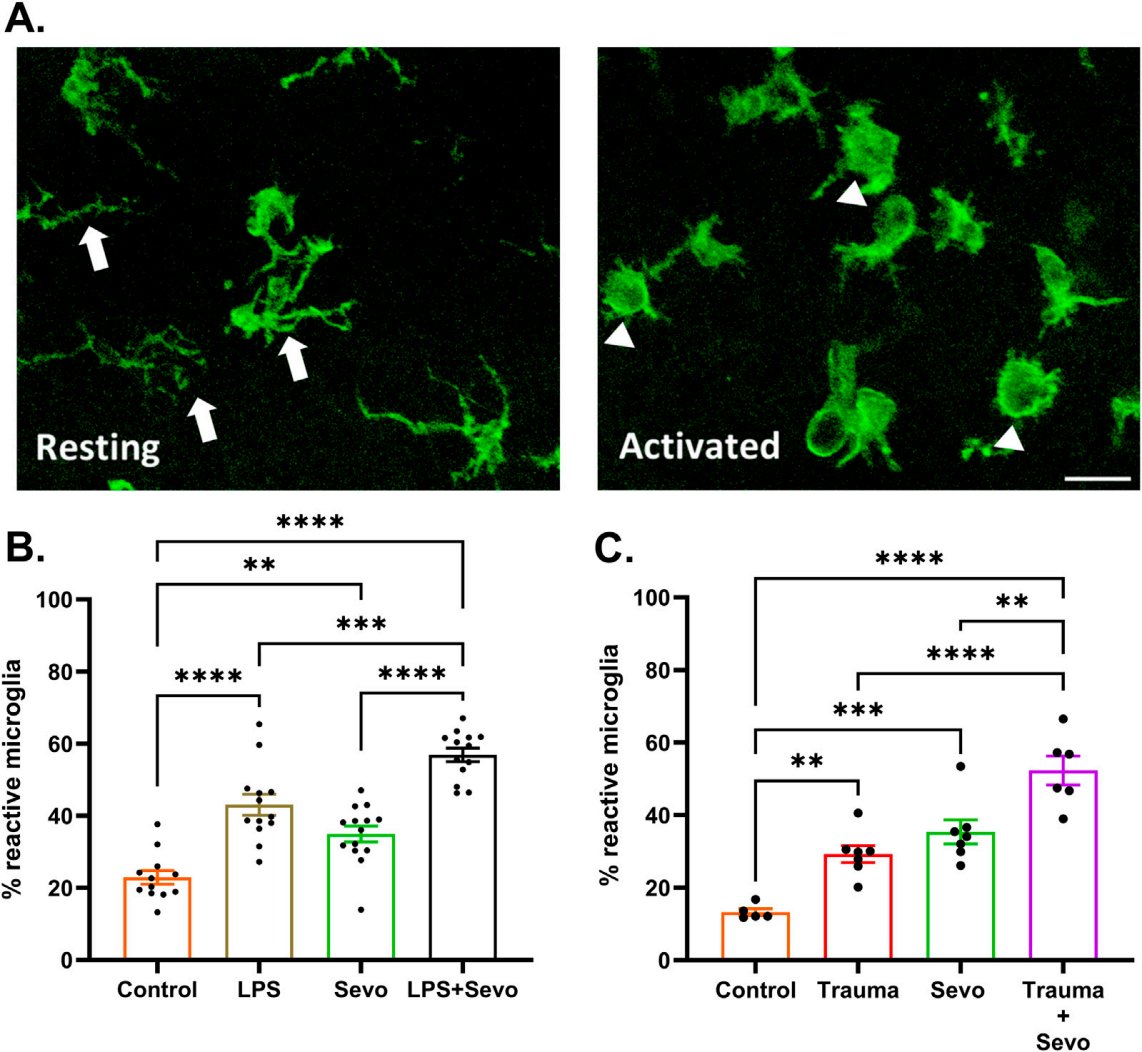
Sevoflurane exposure causes microglia activation in the hippocampal subiculum of PND7 rat pups that is worsen in the setting of trauma- and LPS-induced systemic inflammation. **(A)** Resting microglia (left panel) are ramified with processes (arrows) that probe the extracellular environment and become amoeboid (right panel) in activated state (arrowheads). ×60 magnification, scale bar 20 μm. **(B)** At 2 h post-sevoflurane exposure we detect significant (∼2-fold) increase in microglia activation in LPS or sevoflurane alone groups in the subiculum compared to their respective controls. When sevoflurane was administered following LPS, we report a further increase in microglial activation (∼3-fold above controls). **(C)** At 2 h post-sevoflurane exposure we detect significant (∼2-fold) increase in microglia activation in trauma (tibia fracture) or sevoflurane alone groups in the subiculum compared to their respective controls. When sevoflurane was administered following trauma, we report a further increase in microglial activation (∼4.5-fold above controls). Because there were no gender difference in microglia activation densities, the results from both sexes are combined. One-way ANOVA with Tukey’s *post hoc*. **p < 0.01, ***p < 0.001, ****p < 0.0001.

In view of the observed intensity and duration of sevoflurane-induced neuroapoptosis in the setting of systemic inflammation, we set out to examine the role of microglia since they serve as the resident macrophages within the brain and respond to stress, typically triggering and propagating a pro-inflammatory state. Microglia are ramified with processes that probe the extracellular environment and become amoeboid with retracted processes in an activated state (Figure 4A). We assessed microglia activation using morphological features of Iba1^+^ labelled microglia in the hippocampal subiculum. Our data collected 2 h after sevoflurane exposure demonstrate that LPS, trauma (tibial fracture), or sevoflurane alone can independently cause a ∼2-fold increase in microglia activation in the subiculum compared to their respective controls. When sevoflurane was administered following LPS (Figure 4B) or trauma (Figure 4C), a further increase in microglial activation was observed (∼3- and ∼4.5-fold above controls, respectively; p < 0.0001).

**FIGURE 5:**
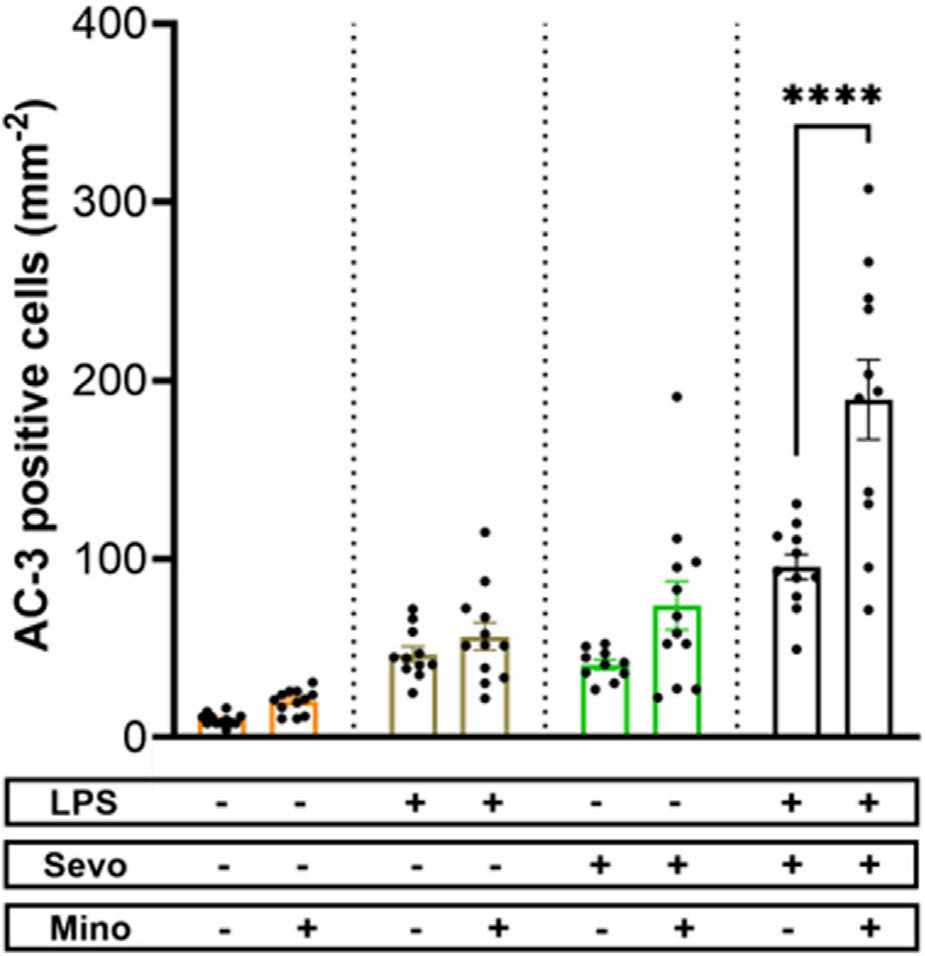
Microglia inhibition with minocycline results in worsening of sevoflurane-induced neuroapoptosis in the setting of LPS-induced systemic inflammation. When PND7 rat pups were treated with minocycline (Mino) at the time of LPS administration and again 12 h later at the time of sevoflurane (sevo) exposure we found that the density of AC3^+^ cells evaluated 2 hours post-sevoflurane was significantly increased in neuroapoptosis compared to those without minocycline. The difference between minocycline-pretreated groups and other animal groups (controls, LPS alone, sevoflurane alone) was not significant. Because there were no gender difference in microglia activation densities, the results from both sexes are combined. Two-way ANOVA with Sidak’s *post hoc*. ****p < 0.0001.

**FIGURE 6:**
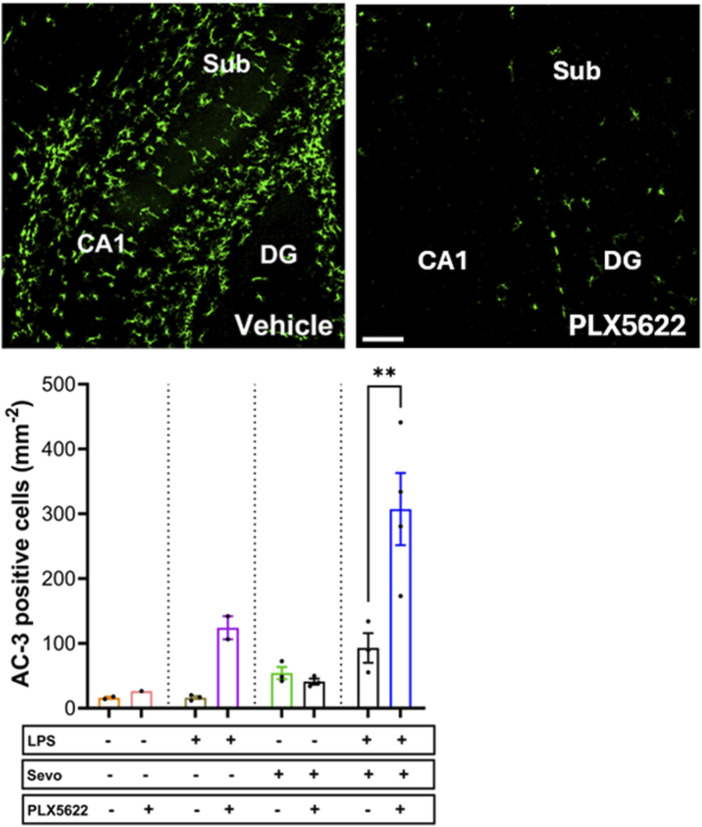
Microglia depletion with PLX5622 results in worsening of sevoflurane-induced neuroapoptosis in the setting of LPS-induced systemic inflammation. When PND7 rat pups were treated with a more selective microglia inhibitor, PLX5622 at the time of LPS administration and again 12 h later at the time of sevoflurane exposure, we found that the density of AC3^+^ cells evaluated 2 hours post-sevoflurane was significantly increased compared to vehicle-treated counterparts. The difference between PLX5622-pretreated animals and corresponding vehicle-treated animals within groups (controls, LPS alone, sevoflurane alone) was not significant. Because there were no gender difference in microglia activation densities, the results from both sexes are combined. ×10 magnification, scale bar 150 μm. Two-way ANOVA with Sidak’s *post hoc*, **p < 0.01.

Considering that the pattern of microglia activation (Figure 4) mimics the pattern of neuroapoptosis (Figure 1) we set out to further probe whether activated microglia may be, at least in part, responsible for amplified sevoflurane neurotoxicity in the setting of systemic inflammation. We exposed PND7 rat pups to minocycline to block microglial activation [36, 37] at the time of LPS administration and again 12 h later at the time of sevoflurane exposure. AC3^+^ cells were evaluated 2 h post-sevoflurane exposure. We observed significant increase in neuroapoptosis in minocycline-treated pups exposed to LPS + sevo compared to those without minocycline (p < 0.0001) (Figure 5). Considering that minocycline is not selective for microglia but can also downregulate other cell types (e.g., T-lymphocytes), we repeated this experiment with a more selective microglia-depleting agent, PLX5622. PLX5622 was administered once daily for 3 consecutive days to deplete the microglia prior to LPS administration and again 12 h later at the time of sevoflurane exposure. This treatment resulted in almost complete removal of microglia as shown in the representative microphotographs (Figure 6). AC3^+^ cells were quantified 2 h post sevoflurane exposure. Similarly to minocycline (Figure 5), we observed a significant increase in neuroapoptosis in PLX5622-treated pups exposed to LPS + sevo compared to those not pretreated with PLX5622 (p < 0.01) (Figure 6). These findings would suggest that microglia in the inflammatory setting is very important for cleaning damaged neurons demonstrating that their loss could result in the accumulation of neuronal debris and further promulgation of the inflammatory responses and enhanced neuronal damage.

**FIGURE 7:**
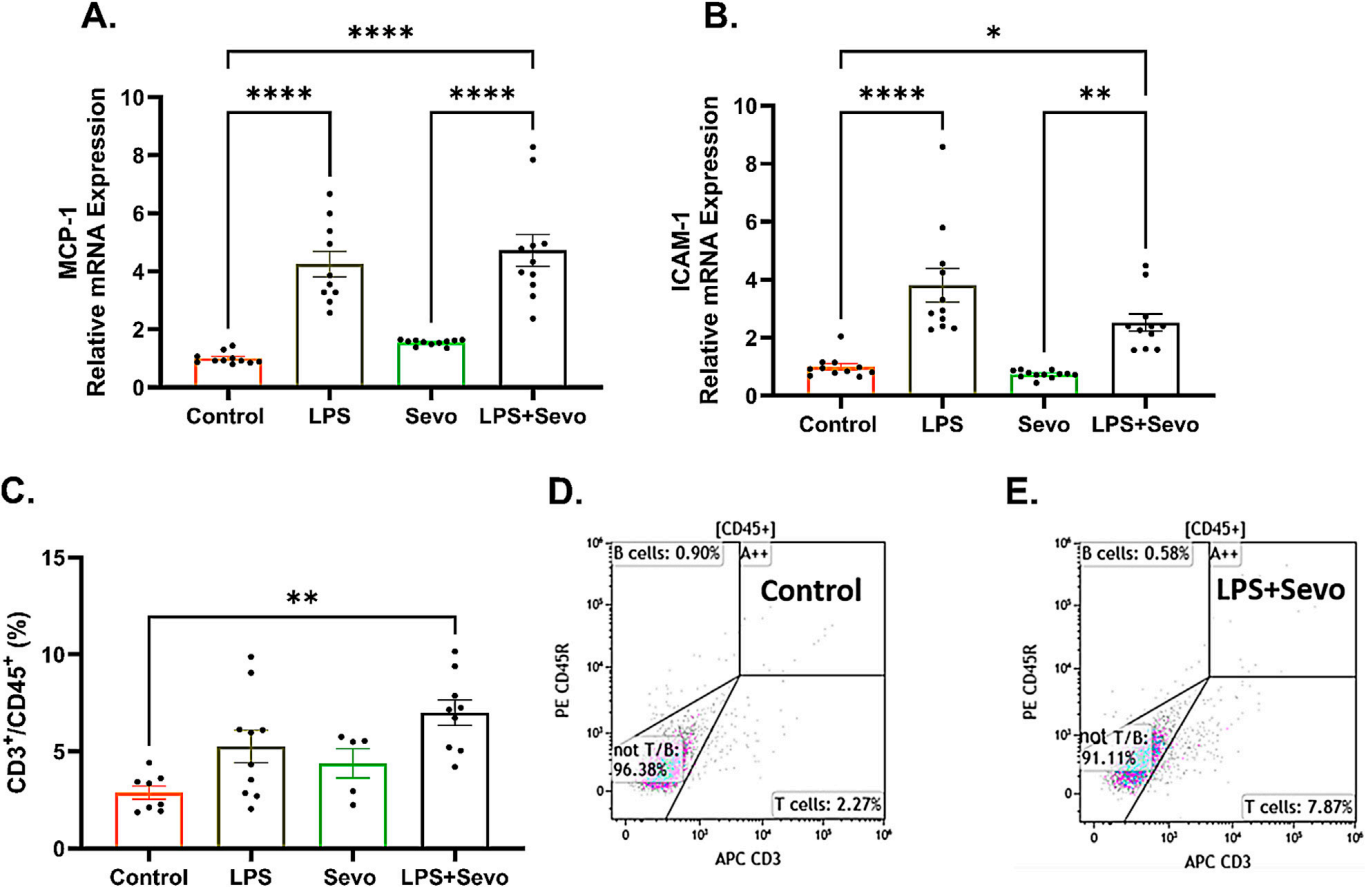
Sevoflurane exposure in the setting of LPS-induced systemic inflammation causes a significant increase in microglia-secreted cytokine, MCP-1 and cell adhesion molecule, ICAM-1 resulting in T-lymphocyte infiltration in hippocampal subiculum of PND7 rat pups. **(A)** There is a significant upregulation MCP-1 mRNA in LPS + sevo group when compared to controls and sevo-alone groups. **(B)** Similarly, we found that, when compared to controls and sevo alone groups, there is a significant upregulation of ICAM-1 mRNA expression in LPS + sevo group. **(C)** There is a significant increase in the proportion of T-lymphocytes in the hippocampus of LPS + sevo rat pups compared to controls. **(D)** Representative flow scatter plots in control group. **(E)** Representative flow scatter plots in sevoflurane group in the setting of systemic inflammation. Because there were no gender difference, the results from both sexes are combined. One-way ANOVA with Tukey’s *post hoc*. *p < 0.05, **p < 0.01, ****p < 0.0001.

We have previously published that pro-inflammatory cytokines, IL-1β and possibly IL-18 are upregulated in the brains of the animals exposed to systemic inflammation [12]. To further probe the role of proinflammatory cytokines, we examined the levels of MCP-1, known to upregulate cell adhesion molecules that allow peripheral immune cells to enter the brain [21, 24]. In the same vein we examined the expression of ICAM-1, a cell adhesion molecule and transmembrane glycoprotein that could be induced by upregulated MCP-1. We observed a significant 4.7-fold increase in MCP-1 (Figure 7A) and 2.5-fold increase in ICAM-1 mRNA expressions (Figure 7B) (p < 0.05) in LPS + sevo treated rats compared to controls.

Since upregulated MCP-1 promotes T-lymphocyte infiltration, we decided to determine the presence of peripheral immune cells in sevoflurane exposed rat pups in the setting of LPS-induced systematic inflammation using flow cytometry. To minimize the risk of contamination from within the traversing blood vessels, animals were thoroughly perfused with ice-cold PBS immediately prior to hippocampal dissection. As shown in Figure 7C, we found a significant increase (p < 0.01) in the proportion of T-lymphocytes in the hippocampus of LPS + sevo rat pups compared to controls (representative flow scatter plots are shown in Figures 7D,E). No differences in proportions of B-cells or neutrophils were observed in our CD45^+^ population (data not shown). Based on this finding we suggest that sevoflurane anesthesia in the setting of systemic inflammation results in significant T-lymphocyte infiltration in hippocampal subiculum.

## Discussion

**FIGURE 8:**
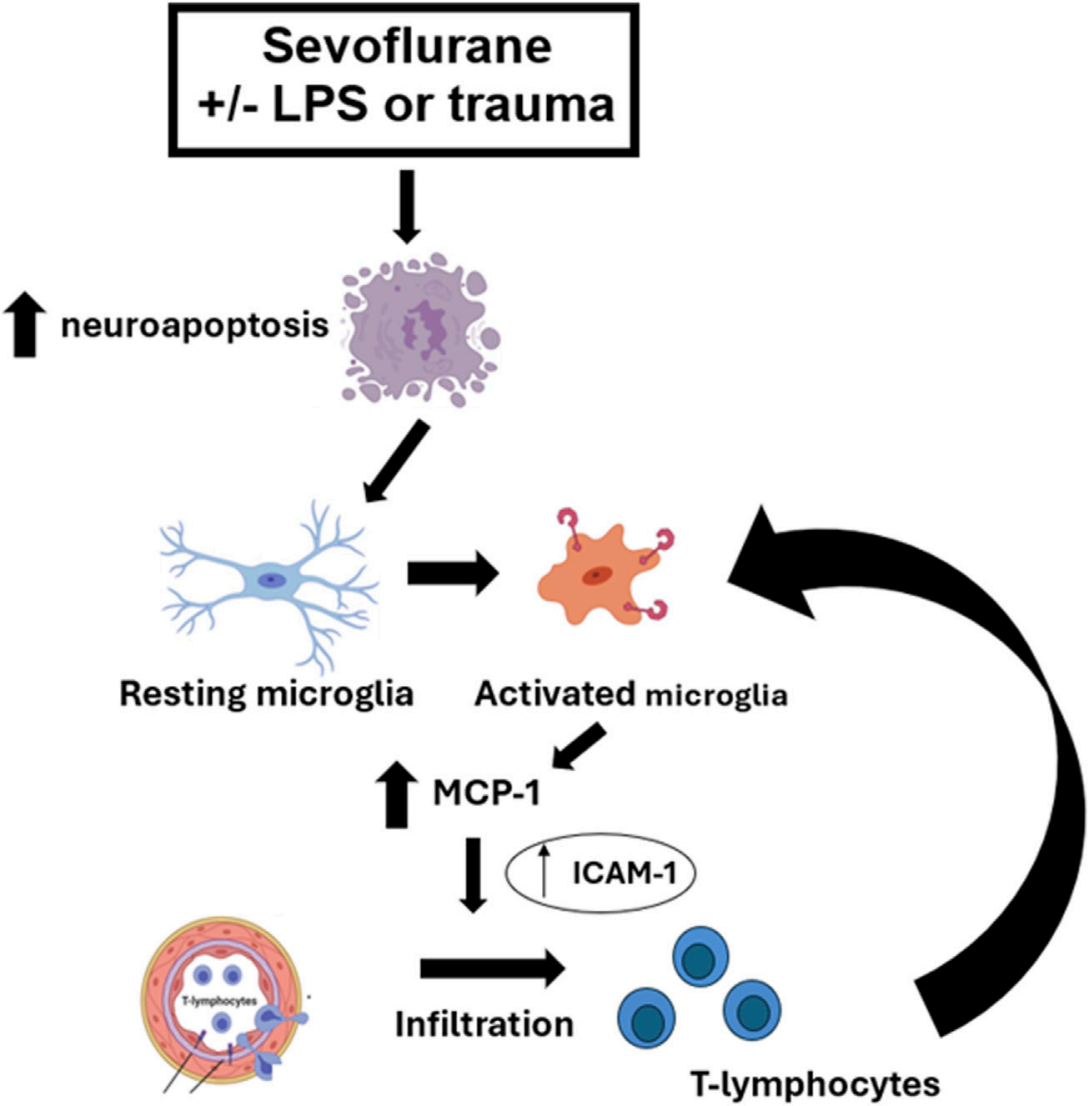
Schematic diagram of the proposed pathway in the setting of sevoflurane-induced systemic inflammation-propagated developmental neuroapoptosis in the hippocampal subiculum of PND7 rat pups. Sevoflurane-induced neuroapoptosis triggers activation of neuroprotective microglia, which in turn releases proinflammatory cytokine, MCP-1 and activates endothelial cell adhesion molecule, ICAM-1. This leads to T-lymphocyte infiltration in the subiculum, an event that further propagates neuroprotective microglia activation in an attempt to control neuroapoptosis, clear the debris and restore the neuropil.

Here we show that an early exposure to sevoflurane during critical stages of synaptogenesis leads to significant neuroapoptosis in young rat pup subiculum, which is worsened in the setting of systemic inflammation caused by either LPS injection or trauma (tibial fracture). The worsening is not only shown in terms of the intensity of neuroapoptosis but in its duration and onset. Our mechanistic studies presented herein suggest that sevoflurane-induced neuroapoptosis triggers activation of microglia, which in turn leads to the upregulation of proinflammatory cytokine MCP-1 and endothelial cell adhesion molecule, ICAM-1 mRNA levels in the hippocampus. This results in T-lymphocyte infiltration in the hippocampal subiculum, an event that further perpetuates microglial activation in an attempt to control neuroapoptosis which is suggested by the fact that microglia depletion leads to a significant worsening of sevoflurane-induced developmental neuroapoptosis. The series of proposed events is depicted in Figure 8.

Due to its complexity, the studies assessing the role of inflammation in anesthesia-induced developmental neurotoxicity have been limited and sometimes at odds with each other [38] with some reporting the worsening of neurotoxicity and long-lasting impairment of cognitive function [12, 39] and other showing amelioration of anesthesia neurotoxicity [40, 41]. Most recent report indicates that sevoflurane, in the setting of LPS-induced systemic inflammation, causes significant upregulation of activated caspase-1 and NLRP1 (NLR family pyrin domain containing 1) along with related proinflammatory cytokines, IL-1β, and IL-18 [12]. This was suggested to result in the formation of caspase-1/NLRP1 inflammasome complex which in turn activates caspases-9 and -3 thus further propagating sevoflurane-driven neuronal demise [12]. Importantly, it also resulted in worsening of learning and memory deficits in male rodents and heightened anxiety-related behavior in female rodents, a sex-specific change noted when tested in young adulthood [12]. As complex as the phenomenon may be, it is becoming more apparent that systemic inflammation could be detrimental to anesthesia-induced developmental neurotoxicity thus suggesting that studying clinically relevant conditions of a disease is paramount. Our work presented herein would suggest that similarly to systemic inflammation caused by LPS, a seemingly regional inflammation caused by bone injury, namely, tibial fracture, followed by surgical repair, using open reduction and internal fixation approach, could result in very similar neurotoxic effects in very young brain.

Based on currently available knowledge which suggests that cytokines and inflammasomes play an important role in the setting of systemic inflammation, we reasoned that microglia could play an important role as well. As specialized macrophages, microglia is considered the innate immune cells in the brain responsible for neuronal protection against different pathogens [13–16, 42, 43]. Through their phagocytic activity microglia control neuronal pruning and the refinement of neuronal circuitries and as such, they play an important role in brain development since they control every important aspect of synaptogenesis [44–49]. The complex role of activated microglia includes not only promotion of inflammation and neurotoxicity (neurotoxic phenotype) [50, 51] but depending on the activation stimulus, they may promote anti-inflammatory and neuroprotective effects (neuroprotective phenotype) [52]. It has been reported that, as the first line of defense, neurotoxic microglia release pro-inflammatory factors with potentially neurotoxic effects. Along those lines, we have previously reported the upregulation of pro-inflammatory cytokines, IL-1β and IL-18 immediately post-sevoflurane exposure in the setting of LPS-induced systemic inflammation [12]. However, if only neurotoxic phenotype gets activated in this setting, one would expect that global microglia inhibition post-minocycline or depletion post-PLX5622 pretreatment would result in ameliorated caspase-3 activation and not enhanced one as we report herein. This prompted us to hypothesize that neuroprotective subset of microglia which suppress inflammation and promote repair of damaged neuropil [53–55] are important in controlling sevoflurane-induced neuroapoptosis in the setting of systemic inflammation. The suggested mechanism supported by our findings would indicate that the observed rise in activated microglia we report herein could be, at least in part, due to an increase in a subset of neuroprotective microglia in the setting of sevoflurane-induced inflammation-propagated developmental neurotoxicity. It has been reported that a conversion of microglia to neuroprotective phenotype gives rise to so-called ‘acute central inflammation’ deemed to be neuroprotective due to its clearing and repairing properties [13, 56]. The puzzling question though remains whether microglia inhibition with minocycline or depletion with PLX5622 targets neuroprotective microglial phenotype more so than the neurotoxic one thus disturbing the fine balance between microglia-dependent neurotoxic and neuroprotective pathways leading to a more substantial neuronal damage post-sevoflurane treatment in the inflammation setting. Further detailed analysis of neurotoxic and neuroprotective microglial phenotype and their interplay in this experimental setting would shed more light on this interesting proposition.

Microglia are instrumental in cleaning up damaged neurons and are considered to be irreplaceable in maintaining neuronal health and timely synaptic development [44–49]. Because of the substantial crosstalk and synergistic activity between microglia and adaptive immune system components during inflammatory conditions [57], an important consideration in this setting is the interaction between T-lymphocytes and neuroprotective microglia. It has been reported that neuroprotective microglia promote local infiltration of T-lymphocytes by expressing MHC-I (major histocompatibility complex-I) [58, 59] and by releasing neuroprotective cytokines such as IL-4, -10 and −13, TGF-β and IGF-1 in the local milieu [55, 60]. Importantly, this in turn can induce the differentiation of naïve T-cells to neuroprotective Th2 and Treg T-cell phenotypes which can release their own anti-inflammatory cytokines, IL-4, -10 and −13 and promote further T-cell infiltration while decreasing the conversion to neurotoxic microglia and reducing the activation of pro-inflammatory type T-cells [57, 61]. Although not designed to examine such complex interaction between infiltrated T-lymphocytes and their various phenotypes on the one side with neuroprotective microglia on the other, our results would suggest that local infiltration of T-cell (but not B-cells), is important in controlling neuroprotective microglia activation and neuronal demise in the setting of sevoflurane-induced neuroapoptosis. This notion is supported by our findings that microglia depletion results in worsening of neuronal damage, most likely due to a disturbed feedback loop between microglia activation and T-lymphocyte infiltration known to play an important role in microglia phagocytosis which improves the clearing of the debris and repairing of damaged neuropil [57, 61]. More detailed assessment of T-cell and microglia phenotypes and secreting properties would be necessary to examine this view. It is worth noting that our work presented herein was focused on the acute timeline, i.e., at 17 h after the initiation of inflammation and only 2 h after the end of sevoflurane exposure. Although this is the optimal time for the assessment of the severity of neuroapoptosis, it is relatively early for detection of T-cell infiltration and its phenotype conversion [16]. Based on previously published work, it appears that later timepoints would be useful in assessing the full scope of T-cells and microglia interactions in the setting of sevoflurane-induced inflammation-propagated neuronal demise.

In the attempt to make our animal work more relevant to the clinical setting and hence more translational, we introduce for the first time a model of trauma in very young animals that is caused by bone fracture. Since the vulnerability to GA-induced developmental neurotoxicity has been reported in pediatric groups up to 4 years of age [62–67], we believe that using orthopedic trauma model is relevant since orthopedic injuries occur in that age group [68, 69] in otherwise healthy and active children. A trauma model of tibial fracture simulates aseptic inflammation since it entails a small skin nick, blunt manipulation of soft tissue and tibial fracture followed by internal fixation, the components relevant to trauma-like disease state [10, 11]. In view of our findings that trauma-induced propagation of sevoflurane-induced neurotoxicity mimics one described with well-known model of systemic inflammation caused by LPS [12, 16, 20], we believe that tibia fracture is a promising model that could get us a step closer to improving our understanding of a very complex phenomenon of anesthesia-induced developmental neurotoxicity in the setting of a disease. This is vitally important considering that exposure to anesthesia is exceedingly rare in the absence of any kind of pathological process.

While GA-induced developmental neurotoxicity was greatly amplified after orthopedic trauma compared to sham procedure, in our studies the insult of trauma alone was not sufficient to induce quantifiable degree of neuroapoptosis, and the reasons behind this observation are not entirely clear. We propose that the systemic inflammation in the case of tibia fracture is not as robust as what has been reported after the LPS injection. Furthermore, LPS easily crosses blood-brain barrier and is well-known as a powerful inducer of not only systemic and but neuroinflammation as well. Hence, LPS alone is capable of initiating neuroapoptotic cascade of events through massive release of inflammatory cytokines and accumulation of reactive oxygen species in the brain which in turn activates caspases-8, -9, and -3. Trauma, on the other hand, relies on circulatory systemic inflammatory markers to initiate a neuroinflammation. Hence, it appears that while effective at priming the intracellular inflammatory machinery, a second signal provided by the GA is needed to trigger the full scope of GA-induced, inflammation-amplified developmental neuroapoptosis [12, 70, 71].

In conclusion, our work presented herein suggests that sevoflurane neurotoxicity is enhanced in the setting of systemic inflammation in terms of its onset, the intensity and duration that could, at least in part, be explained by a complex interplay between microglia activation and T-cell infiltration.

## Data Availability

The raw data supporting the conclusions of this article will be made available by the authors, without undue reservation.
